# *Helicobacter pylori cag*-Pathogenicity Island-Dependent Early Immunological Response Triggers Later Precancerous Gastric Changes in Mongolian Gerbils

**DOI:** 10.1371/journal.pone.0004754

**Published:** 2009-03-09

**Authors:** Tobias Wiedemann, Eva Loell, Susanna Mueller, Mechthild Stoeckelhuber, Manfred Stolte, Rainer Haas, Gabriele Rieder

**Affiliations:** 1 Max-von-Pettenkofer-Institute for Hygiene and Medical Microbiology, Ludwig Maximilians University, Munich, Germany; 2 Institute of Pathology, Ludwig Maximilians University, Munich, Germany; 3 Institute of Anatomy, Ludwig Maximilians University, Munich, Germany; 4 Institute of Pathology, Klinikum Bayreuth GmbH, Bayreuth, Germany; University of Hyderabad, India

## Abstract

Infection with *Helicobacter pylori*, carrying a functional *cag* type IV secretion system (*cag*-T4SS) to inject the Cytotoxin associated antigen (CagA) into gastric cells, is associated with an increased risk for severe gastric diseases in humans. Here we studied the pathomechanism of *H. pylori* and the role of the *cag*-pathogenicity island (*cag*-PAI) for the induction of gastric ulcer and precancerous conditions over time (2–64 weeks) using the Mongolian gerbil model. Animals were challenged with *H. pylori* B128 (WT), or an isogenic B128Δ*cagY* mutant-strain that produces CagA, but is unable to translocate it into gastric cells. *H. pylori* colonization density was quantified in antrum and corpus mucosa separately. Paraffin sections were graded for inflammation and histological changes verified by immunohistochemistry. Physiological and inflammatory markers were quantitated by RIA and RT-PCR, respectively. An early *cag*-T4SS-dependent inflammation of the corpus mucosa (4–8 weeks) occurred only in WT-infected animals, resulting in a severe active and chronic gastritis with a significant increase of proinflammatory cytokines, mucous gland metaplasia, and atrophy of the parietal cells. At late time points only WT-infected animals developed hypochlorhydria and hypergastrinemia in parallel to gastric ulcers, gastritis cystica profunda, and focal dysplasia. The early *cag*-PAI-dependent immunological response triggers later physiological and histopathological alterations towards gastric malignancies.

## Introduction

The bacterial pathogen *Helicobacter pylori* colonizes the human gastric mucosa of about 50% of the world population to induce a chronic gastritis. As a consequence of an inflammation lasting for decades, gastric sequelae are developed like peptic ulcer and malignant diseases, such as gastric adenocarcinoma or MALT (mucosa-associated lymphoid tissue)-lymphoma [1], [2]. Due to epidemiological studies the WHO declared *H. pylori* as a class I carcinogen in 1994 [3]. Furthermore, a *H. pylori*-induced chronic inflammation in the human body represents a risk factor for developing gastric cancer *per se*. A malignant transformation of cells is usually a multi-factorial process [4], [5], which is also true for gastric carcinoma. In addition to *H. pylori* infection, environmental (diet, smoking) [6] and host factors (gene polymorphisms, e.g. interleukin (IL)-1β) [7] are certainly involved in its induction. Therefore the question remains what is the contribution of *H. pylori* for induction of gastric cancer.

*H. pylori* produces a number of important virulence factors inducing a local inflammation in the stomach. Two major virulence factors have been studied intensively, the vacuolating cytotoxin A (VacA) [8] and the cytotoxin-associated antigen A (CagA). VacA is a secreted toxin that induces vacuoles in gastric epithelial cells, modulates cellular permeability, and enters immune cells via the β2 integrin receptor [8]. This is a possible mechanism for *H. pylori* to escape the adaptive immune system establishing a chronic inflammation. The *cag*-type IV secretion system (*cag*-T4SS), which is encoded on the *cag*-pathogenicity island (*cag*-PAI) [9] injects the effector protein CagA as well as peptidoglycan [10], resulting in activation of nuclear factor (NF)-κB and gastric inflammation. Translocated CagA is phosphorylated on certain tyrosine residues, leading to actin-cytoskeletal rearrangements, elongation, and the scattering phenotype of infected cells *in vitro*. The “needle”-like structure of the T4SS interacts with the integrin α5β1-receptor on the gastric epithelial cells, to deliver CagA into the host cells [11], which finally leads to an activation of signal cascades inducing pro-inflammatory cytokines. Those *H. pylori* strains that express VacA and carry a complete and functional T4SS to translocate CagA into gastric host cells are designated as type I-strains, whereas type II-strains are defective in the *cag*-PAI and do not secrete functional VacA. Epidemiological studies have shown that type I-strains are associated with a more pronounced development of peptic ulcer and gastric cancer in humans [12], [13].

To study the effect of *H. pylori* on the induction of gastroduodenal diseases different animal models have been established. *H. pylori* type I-strains are not fully virulent in mouse models, since they neither inject CagA, nor does VacA induce immunomodulation in murine T cells [8]. The mouse model is limited, since it cannot be used to recapitulate the *cag*-PAI dependent gastric carcinogenesis. It could be shown that infection of wild-type mice with *cag* ^+^ strains frequently leads to deletions within the *cag*-PAI [14], [15]. Therefore, the Mongolian gerbil model has been established to study *H. pylori* pathogenesis towards gastric adenocarcinoma [16].

In earlier studies analyzing only a single time point of infection (seven month) we could demonstrate that only a chronic infection of *H. pylori* type I-strain was able to induce an atrophic corpus-dominant gastritis in Mongolian gerbils [17], which is a risk factor for developing gastric cancer. This observation was supported by human studies to be a precancerous condition, essential to be followed up tightly.

To gain more insight into the pathomechanisms of *H. pylori* and the role of the *cag*-PAI on the development of gastroduodenal diseases, we now performed a time course experiment. Mongolian gerbils were infected for 2, 4, 8, 16, 32, and 64 weeks with *H. pylori* B128 WT- (type I), or B128 Δ*cag*Y mutant-strain (type II). Drastic differences were observed in the capacity of the B128 WT and the Δ*cag*Y-mutant strain to colonize the gastric antrum and corpus mucosa inducing severe inflammation over time.

Our data suggest that an early inflammation in the antrum and especially in the corpus mucosa at eight weeks of infection, which is a *cag*-PAI-dependent mechanism, is triggering several months later physiological (hypochlorhydria and hypergastrinemia) and histopathological changes towards precancerous conditions. In general, our long-term *in vivo*-study reveals early markers for late gastric diseases that represent potential risk factors for precancerous transformation with a possible clinical application.

## Materials and Methods

### Bacterial strains

*Helicobacter pylori* B128, a Mongolian gerbil-adapted type I-strain (CagA, VacA: s1m2) [18], and its isogenic mutant B128Δ*cag*Y (both streptomycin resistant) were used in this study as previous described [17]. Both strains carried a chromosomal streptomycin resistance that allowed quantitative recovery of *H. pylori* from the gerbil stomach by antibiotic selection (streptomycin 250 mg/L) [19]. Each antral and corpus tissue specimen was homogenized (glass homogenizer, Ochs, Bovenden, Germany) in 1 ml Brucella broth, appropriate dilutions were spread on selective serum plates (GC agar (Oxoid, Wesel, Germany) supplemented with horse serum (8%), vancomycin (10 mg/l), trimethoprim (5 mg/l), nystatin (1 mg/l)), and streptomycin (250 mg/l)), and incubated under microaerophilic conditions (85% N_2_, 10% CO_2_, 5% O_2_) at 37°C for up to five days. Numbers of colony forming units (CFU) were expressed per gram of gastric tissue. *H. pylori* reisolates were tested for urease (urea broth, Oxoid), oxidase (DrySlide, BBL), and catalase (3% hyperperoxid-solution) activity.

### Animals and infection experiments

Outbred Mongolian gerbils (n = 167 females) from our own breeding colony were specific pathogen free (SPF) and housed in SEALSAFE IVC cages (H-Temp, Tecniplast, Hohenpeissenberg, Germany) in an air-conditioned biohazard room (room temperature, 23+/−2°C; relative humidity 55+/−5%; 12/12-h light/dark cycle) with free access to a commercial gerbil diet (ssniff Gerbil, SSNIFF, Soest, Germany) and sterile tap water. Animals at the age of 8–12 weeks were challenged orogastrically three-times over five consecutive days with approximately 10^9^ viable *H. pylori*. Age-matched control animals were inoculated with identical volumes of sterile Brucella broth alone. All experiments and procedures carried out were conducted in accordance with the Guidelines for the Care and Use of Laboratory Animals and approved by the Regierung von Oberbayern (AZ 55.2-1-54-2531-41/04 and 55.2-1-54-2531-78/05). The animals were sacrificed after specified time of infection (2, 4, 8, 16, 32, and 64 weeks), the stomach opened along the greater curvature, and the gastric tissue conserved separately in antrum and corpus as previous described [17].

### Histopathology

Paraffin embedded longitudinal sections of antrum and corpus were hematoxilin & eosin (H&E) stained for histomorphologic grading of gastritis and mucosal changes. The activity and chronicity of gastritis (scale 0–3) were analyzed double blind according to the updated Sydney System [20] and the intensity of inflammation, metaplasia, and ulcer development (scale 0–5) according to the grading scheme for rodents by Garhart *et al.* [21]. The presence of mucous gland metaplasia was confirmed by a periodic acid-Schiff (PAS)/Alcian blue stain (pH 2.5). The Cancer Risk Index was applied as previous described [22]. In brief, an increased carcinoma risk was shown if active and chronic gastritis in corpus tissue was greater than in the antrum and if metaplastic changes were present.

### Immunohistochemistry

Parietal cells were detected immunohistochemically by applying anti-proton pump (PP)-antibody (Medical & Biological Laboratories, LTD, Naka-ku Nagoya, Japan). Positive-staining cells were visualized with diaminobenzidine (DAB) (Vectastain Elite ABC Kit; Vector Laboratories) and morphometrically analyzed with MetaMorph (Visitron, Puchheim Germany) software.

The evaluation of PP-positive cells as marker of the atrophy was performed using following score: 0, PP-positive cells distributed as in the non-infected control tissue; 1, small areas lacking PP-positive cells; 2, large areas without PP-positive cells; 3, no PP-positive cells in the entire tissue section.

### pH measurement

The pH value of the gastric mucosa was measured at the corpus tissue using color-fixed indicator test sticks (pH-Fix 0.0–6.0, Macherey-Nagel, Dueren, Germany).

### Gastrin and somatostatin radioimmunoassay (RIA)

Plasma was isolated by centrifugation (9,000 rpm, 15 min at 4°C) from heparinized blood (2 ml) that was collected by cardiac puncture freshly after sacrifice of the animals. For the somatostatin RIA the plasma was extracted with Sep-Pak C18 cartridges (Waters GmbH, Eschborn, Germany). The gastrin- and somatostatin RIA were performed as described in the gastrin [^125^I] (MP Biomedicals, Heidelberg, Germany) and somatostatin [^125^I] (IBL, Hamburg, Germany) radioimmunoassay kits, respectively. Rabbit anti-human-gastrin (G-17) and -somatostatin (synthetic cyclic 14) antibodies in human serum albumin were used. The sensitivity of the gastrin and somatostatin assay was 3.3 pg/ml and 6.0 pg/ml, respectively.

### RNA isolation and real-time RT-PCR

RNA isolation and real-time reverse transcription-polymerase chain reaction (RT-PCR) measurement were applied as described previously [17]. cDNA was synthesised using 1 µg total RNA, random hexamer oligonucleotide primers, and TaqMan Reverse Transcriptase Kit (Roche). Oligonucleotide primer and probes specific for IL-1β, interferon (IFN)-γ, KC, somatostatin (Sst), IL-6, IL-10, tumor necrosis factor (TNF)-α, gastrin, histidine decarboxylase, and the housekeeping gene 18S rRNA ([17] and Table S1) were applied for real-time RT-PCR (ABI PRISM 7000, Applied Biosystems). All data were normalized with the corresponding 18S rRNA transcription level using a comparative delta Ct method.

### Statistical Analysis

The results were statistically analyzed using the Mann-Whitney *U*-test for unpaired groups and the Fischer's Exact Test for inter-group differences. The p value <0.05 was considered as significant.

## Results

### Colonization density *of H. pylori* wild type decreases in antrum and increases in corpus over time

Mongolian gerbils were orogastrically infected with *H. pylori* B128 wild type (WT) or *H. pylori* B128Δ*cag*Y-mutant strains in a time course experiment of 2, 4, 8, 16, 32, and 64 weeks. In WT-infected gerbils a medial reisolation rate of 85% was obtained, whereas all mutant-infected groups showed reisolation rates of 100% (Table 1). The reisolated *H. pylori* were selected by streptomycin to exclude growth of other gastric bacteria.

**Table 1:** Macroscopic and histopathological findings of *Helicobacter pylori*-infected and non-infected Mongolian gerbils.

		2 weeks			4 weeks			8 weeks		
		Non-inf.	WT	Δ*cagY*	Non-inf.	WT	Δ*cagY*	Non-inf.	WT	Δ*cagY*
		(n/n) %	(n/n) %	(n/n) %	(n/n) %	(n/n) %	(n/n) %	(n/n) %	(n/n) %	(n/n) %
Number of animals		6	9	4	8	12	10	5	7	9
Reisolation rate		(0/6) 0	(9/9) 100	(4/4) 100	(0/8) 0	(9/12) 75	(10/10) 100	(0/5) 0	(6/7) 86	(9/9) 100
Lymphoid aggregates	antrum	(0/6) 0	(0/9) 0	(0/4) 0	(0/8) 0	(1/9) 11	(0/10) 0	(0/5) 0	(6/6) 100	(5/9) 55
	corpus	(0/6) 0	(0/9) 0	(0/4) 0	(0/8) 0	(0/9) 0	(0/10) 0	(0/5) 0	(6/6) 100	(1/9) 11
Erosion		(0/6) 0	(0/9) 0	(0/4) 0	(0/8) 0	(1/9) 11	(0/10) 0	(0/5) 0	(6/6) 100	(4/9) 44
Ulcer		(0/6) 0	(0/9) 0	(0/4) 0	(0/8) 0	(0/9) 0	(0/10) 0	(0/5) 0	(1/6) 17	(0/9) 0
Atrophy corpus^A^		(0/6) 0	(0/9) 0	(0/4) 0	(0/8) 0	(0/9) 0	(0/10) 0	(0/5) 0	(6/6) 100	(4/9) 44
Metaplastic changes		(0/6) 0	(0/9) 0	(0/4) 0	(0/8) 0	(0/9) 0	(0/10) 0	(0/5) 0	(6/6) 100	(4/9) 44
Gastritis cystica profunda		(0/6) 0	(0/9) 0	(0/4) 0	(0/8) 0	(0/9) 0	(0/10) 0	(0/5) 0	(1/6) 17	(0/9) 0
Focal dysplasia		(0/6) 0	(0/9) 0	(0/4) 0	(0/8) 0	(0/9) 0	(0/10) 0	(0/5) 0	(0/6) 0	(0/9) 0
Increased carcinoma risk		(0/6) 0	(0/9) 0	(0/4) 0	(0/8) 0	(0/9) 0	(0/10) 0	(0/5) 0	(0/6) 0	(0/9) 0

		16 weeks			32 weeks			64 weeks		
		Non-inf.	WT	Δ*cagY*	Non-inf.	WT	Δ*cagY*	Non-inf.	WT	Δ*cagY*
		(n/n) %	(n/n) %	(n/n) %	(n/n) %	(n/n) %	(n/n) %	(n/n) %	(n/n) %	(n/n) %
Number of animals		5	11	11	9	19	13	8	12	9
Reisolation rate		(0/5) 0	(10/11) 91	(11/11) 100	(0/9) 0	(17/19) 89	(13/13) 100	(0/8) 0	(8/12) 66	(9/9) 100
Lymphoid aggregates	antrum	(0/5) 0	(10/10) 100	(9/11) 82	(0/9) 0	(17/17) 100	(13/13) 100	(0/8) 0	(8/8) 100	(9/9) 100
	corpus	(0/5) 0	(7/10) 70	(0/11) 0	(0/9) 0	(15/17) 88	(4/13) 30	(0/8) 0	(8/8) 100*	(3/9) 33
Erosion		(0/5) 0	(10/10) 100	(3/11) 27	(0/9) 0	(17/17) 100	(6/13) 46	(0/8) 0	(8/8) 100	(8/9) 89
Ulcer		(0/5) 0	(3/10) 30	(0/11) 0	(0/9) 0	(8/17) 47	(0/13) 0	(0/8) 0	(6/8) 75*	(0/9) 0
Atrophy corpus^A^		(0/5) 0	(10/10) 100	(0/11) 0	(0/9) 0	(16/17) 94	(2/13) 15	(0/8) 0	(8/8) 100*	(4/9) 44
Metaplastic changes		(0/5) 0	(9/10) 90	(0/11) 0	(0/9) 0	(16/17) 94	(6/13) 46	(0/8) 0	(8/8) 100*	(4/9) 44
Gastritis cystica profunda		(0/5) 0	(0/10) 0	(0/11) 0	(0/9) 0	(4/17) 24	(1/13) 8	(0/8) 0	(6/8) 75*	(4/9) 44
Focal dysplasia		(0/5) 0	(1/10) 10	(0/11) 0	(0/9) 0	(1/17) 6	(0/13) 0	(0/8) 0	(2/8) 25*	(0/9) 0
Increased carcinoma risk		(0/5) 0	(3/10) 30	(0/11) 0	(0/9) 0	(12/17) 71	(1/13) 8	(0/8) 0	(6/8) 75*	(0/9) 0

A parietal cell atrophy.

* significant increase in comparison to mutant-infected group.

**Figure 1:**
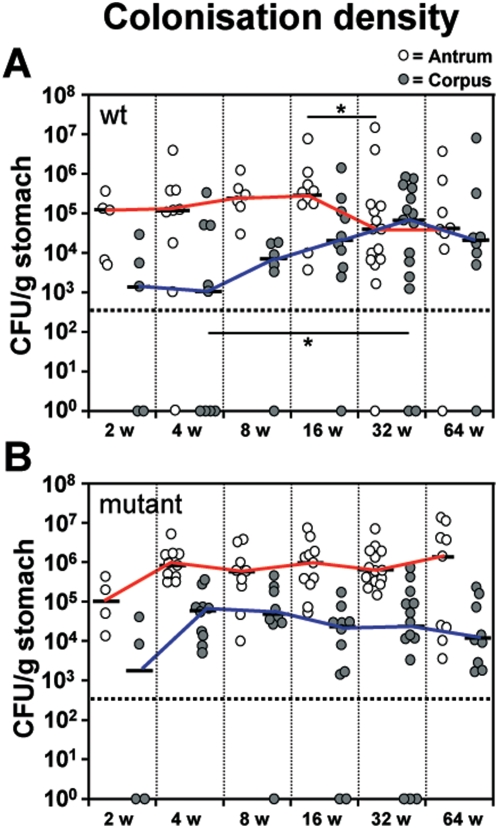
Increased *Helicobacter pylori* B128 WT colonization density shown in corpus mucosa over the time course experiment. Colonization density of antral (white circles) and corpus (gray circles) mucosa in orally challenged gerbils with *H. pylori* B128 WT (A) and B128Δ*cag*Y (B) isogenic mutant strain after 2, 4, 8, 16, 32, and 64 weeks of infection. The interpolated lines connect the medians of the respective time-points. The detection limit was <5×10^2^ colony-forming units (CFU) per gram of stomach (horizontal dotted line). Gastric tissue specimens without *H.pylori* reisolation are shown as null. (*p<0.05).

After two weeks both groups of infected animals started with a comparable colonization density of *H. pylori* in antrum and corpus of 10^5^ and 10^3^ CFU/g stomach, respectively (Figure 1A–B). The B128 WT strain increased its density in the corpus slowly but continuously. After 16 weeks of infection the WT bacteria decreased their number in the antrum and equalized with the corpus colonizing bacteria at 10^4^ CFU/g stomach at 32 weeks of infection. This colonization rate remained stable until 64 weeks of infection (Figure 1A–B). The observed change in colonization density over time is clearly dependent on a functional *cag*-PAI, since the B128Δ*cagY* mutant did not significantly change its bacterial density between 4 and 64 weeks, but maintained a constant difference (1–1.5 log stages) in bacterial load between antral and corpus tissue. It was interesting to observe that the colonization density in the antrum of the mutant-infected gerbils was increased by ≥1 log stage compared to the WT-infected groups.

### An early severe active and chronic gastritis as well as severe histological changes are only induced in wild type infected Mongolian gerbils

**Figure 2:**
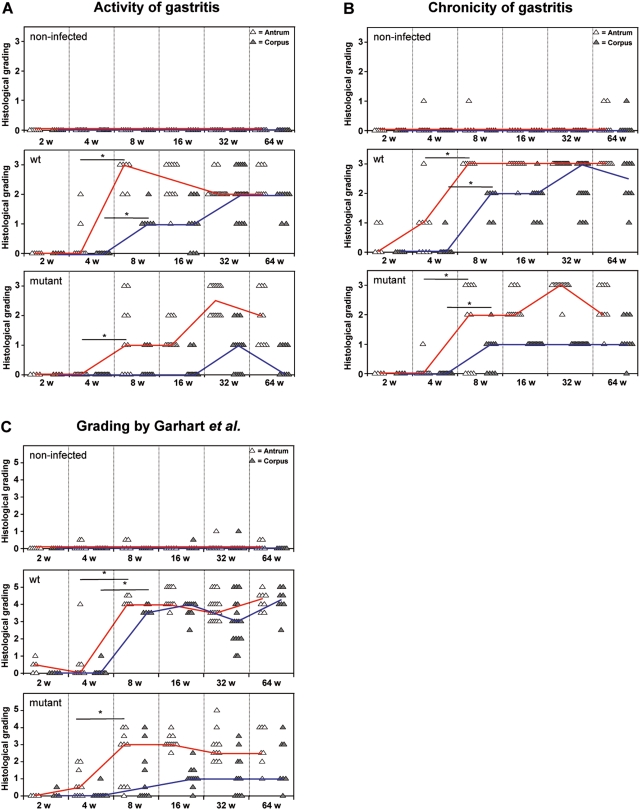
Induction of corpus dominant gastritis is B128 WT dependent. Histological grading of antral (white triangles) and corpus (gray triangles) mucosa. Gerbils were orally challenged with *H. pylori* B128 WT (middle panels) and B128Δ*cag*Y (bottom panels) isogenic mutant strain for 2, 4, 8, 16, 32, and 64 weeks compared with age-matched non-infected controls (upper panels). (A) Active gastritis and (B) chronic gastritis grading was performed according to the upgraded Sydney system, and (C) according to Garhart *et al*. The interpolated lines connect the medians of the respective time-points. (*p<0.05).

To investigate the dynamics of the host response, we analyzed the induction of the innate immune system by *H. pylori*-infection using histology. The grade of active and chronic gastritis in Mongolian gerbils was assessed by the density of neutrophil granulocytes, as well as lymphocytes, macrophages, and plasma cells infiltrating the gastric mucosa, respectively. In WT-infected animals, a severe active and chronic gastritis was observed in H&E stained antral sections at eight weeks of infection (Figure 2A–B). However, in the corpus, active and chronic gastritis gradually increased over time. In contrast to the WT-infected animals, gerbils infected with the B128 Δ*cag*Y mutant revealed an attenuated progress of inflammation in antrum and corpus tissue. Only from 32 weeks of infection onwards the grading value in the antrum of mutant-infected animals reached the level of WT-infected animals (Figure 2A–B).

Applying the grading system by Garhart *et al.* [21], which includes the grade of metaplasia and ulcer development besides the intensity of inflammation, a significant increase at 8 weeks of infection was found in antral and corpus mucosa of WT-infected animals, but not in the corpus of mutant-infected gerbils (Figure 2C).

We conclude that in the antrum an early severe inflammation after 4 weeks of infection is dependent on a functional T4SS, inducing severe histological changes. After a delay of several months a strong antral inflammation is independent of CagA translocation, leading only to slight histological changes. In contrast, a severe inflammation in the corpus is only induced by a *H. pylori* type I-strain. Thus, our data clearly show a different pathomechanism in antrum and corpus mucosa.

### Early precancerous conditions are observed at eight weeks of infection, but only with a *H. pylori* strain able to translocate CagA

**Figure 3:**
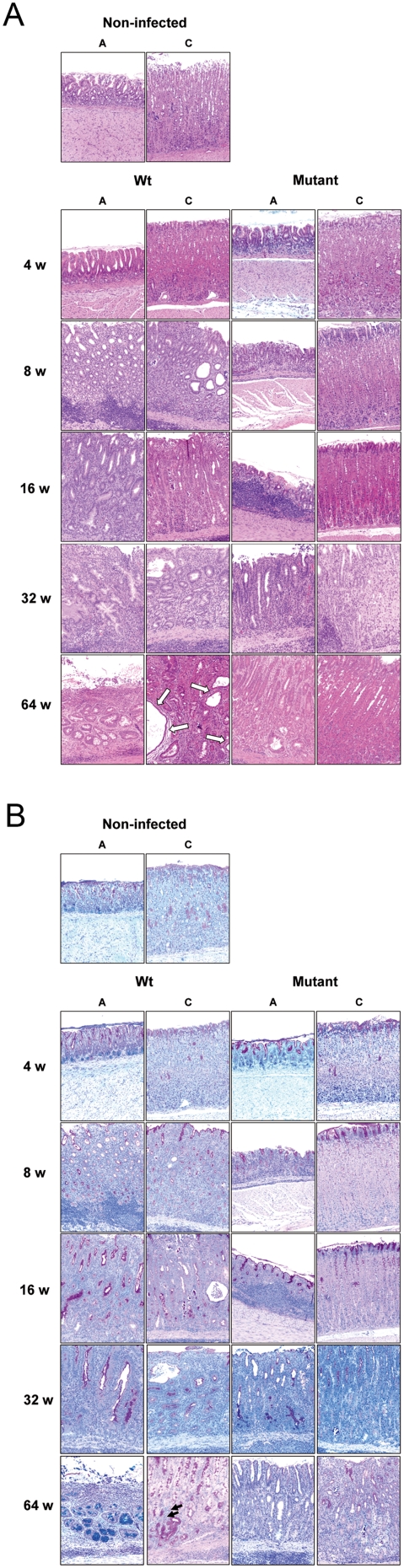
Increased severity of inflammation in *H. pylori* B128 WT infected gerbils. (A) H&E and (B) alcian blue-PAS stained paraffin-embedded antral [A] and corpus [C] tissue (original magnification ×10). Mucous gland metaplasia (black arrows) and gastritis cystica profunda (white arrows) are shown. Gerbils were orally challenged with *H. pylori* B128 WT (left panels) and B128Δ*cag*Y isogenic mutant strain (right panels) for 2, 4, 8, 16, 32 and 64 weeks [w].

Following the sequelae of *H. pylori*-associated chronic gastritis to gastric adenocarcinoma, several histological changes of the mucosa are usually involved, such as atrophy, metaplasia, and dysplasia. By means of H&E as well as Alcian blue/PAS staining, we assessed histopathological changes of the gastric mucosa. The time course experiment revealed an increasing disturbance of the differentiation of the gastric mucosa beginning at 8 weeks of infection with *H. pylori* B128 (Figure 3A). In antrum and corpus of infected gerbils, the severe gastritis is followed by multiple lymphocyte aggregates in mucosa and submucosa, extensive hyperplasia of antral mucosa, a high degree of atrophy (loss of parietal cells) (Figure 3A–B) in the corpus, and metaplastic changes (mucous gland metaplasia) in up to 100% of WT-infected animals. The disturbance of the glandular mucosa is dramatically increasing over the time course in antrum and corpus of WT-infected animals, resulting in the formation of a regenerated epithelium (data not shown), but also a significant increase of gastritis cystica profunda (75%), and focal dysplasia (25%) occurred at 64 weeks of infection (Figure 3A, Table 1). Severe neoplastic changes or gastric adenocarcinoma were not detected in any of the infected animals.

In contrast to WT-infected animals, mutant-infected groups revealed a less dramatic histopathological change of the antral mucosa over time. Although B128Δ*cag*Y colonizes the corpus mucosa very efficiently (Figure 1B), this strain was not able to induce major histopathological changes, since the epithelial and glandular architecture was mainly preserved (Figure 3A, Table 1). Thus, the histopathological changes seen in WT-infected gerbils are dependent on a functional T4SS.

### A changing programme of mucus production in the gastric foveolae and gastric ulcer development due to infection with *H. pylori* type I-strain

By applying the Alcian blue/PAS stain, we determined changes in gastric mucus production during infection, staining paraffin sections red and blue for neutral and acid mucin, respectively (Figure 3B). Beginning at 8 weeks of infection, the neutral mucin secreting cells extended from the epithelium to the isthmus and down to the bottom of the foveolae of WT-infected antral and corpus mucosa (Figure 3B). In parallel, the red staining mucin at the top of the epithelium diminished considerably and the acid mucin secretion at the bottom of the antral glands disappeared entirely at 8 weeks of WT-infected gerbils. In contrast, the mutant-infected animals revealed less pronounced changes of the gastric mucin, indicating that a functional T4SS is important for reprogramming cells in terms of mucin production.

Another sequelae of chronic *H. pylori* gastritis is the development of gastric ulcer. Minor and large erosions of the gastric mucosa, which are precursor lesions of gastric ulcer, were present in 100% of WT-infected animals from 8 weeks onwards. The mutant-infected animals, in contrast, required an additional year of infection, to achieve in 89% of the animals a similar level of erosions (Table 1). Furthermore, gastric ulcers were observed first in WT-infected gerbils after 8 weeks of infection (17%) and increased in frequency up to 75% at 64 weeks of infection. The macroscopic observation of an open stomach of WT-infected gerbils at 64 weeks of infection often showed penetrating ulcers and a loss of the architecture of the gastric mucosa (data not shown). Such pathological changes were not found in any of the mutant- or non-infected animals. Taken together, these observations demonstrate that in contrast to a type II-strain, only a *H. pylori* type I-strain is able to induce severe gastric diseases, such as gastric ulcer or precancerous lesions.

### Atrophy of parietal cells in the corpus is dependent on the *H. pylori* T4SS

**Figure 4:**
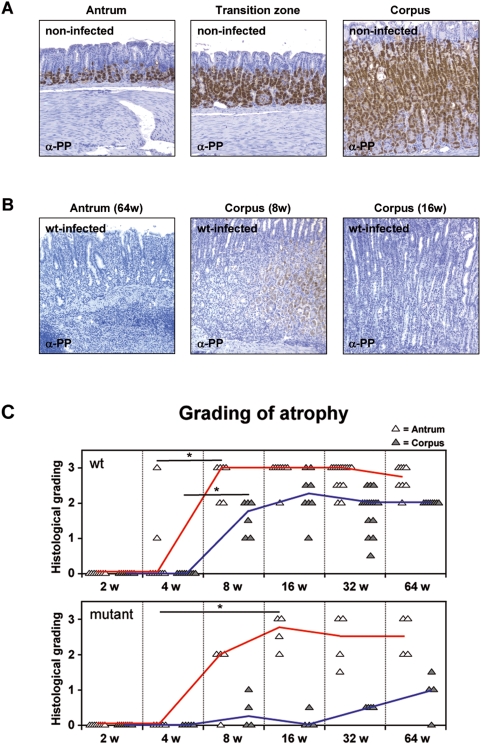
Distribution of parietal cells analyzed by immunohistochemical staining. (A) Parietal cells were detected with anti-proton pump antibody in antral tissue (left panel), transition zone (middle panel), and corpus tissue (right panel) of non-infected Mongolian gerbils (original magnification 10×). (B) Immunohistochemical staining with anti-proton pump antibody detecting complete parietal cell atrophy in antral tissue at 64 weeks (left panel) and beginning at 8 weeks (middle panel) as well as complete parietal cell atrophy in corpus tissue at 16 weeks (right panel) of *H. pylori* B128 WT-infected gerbils. (C) Histological parietal cell atrophy grading of antral (white triangles) and corpus (gray triangles) mucosa infected with *H. pylori* B128 WT (upper panel) and B128Δ*cag*Y isogenic mutant strain (bottom panel) over time. (*p<0.05).

Since atrophy of the glandular mucosa is defined as the loss of parietal cells, we first analyzed the distribution of parietal cells in a horizontal section of the gastric mucosa of a non-infected gerbil stomach by immunohistochemistry. Parietal cells were mainly detected in the corpus but some in the antrum, too (Figure 4A). Therefore, we applied the grading for the atrophy not only to the corpus, but also to the antral tissue. The atrophy was classified into three stages, a beginning, moderate, and complete loss of parietal cells (Figure 4B).

WT-infected gerbils revealed a significantly increased atrophy in antral and corpus mucosa after 4 weeks of infection (Figure 4C). However, mutant-infected animals showed only an atrophy in the antrum tissue but none in the corpus mucosa nearly until the end of the time-course experiment. These data suggest that in the antral mucosa atrophy is induced early after infection in a *cag*-PAI-independent manner, whereas the loss of huge numbers of parietal cells in the corpus is mainly dependent on the activity of the *cag*-T4SS of *H. pylori*.

### Type I *H. pylori* increases the Cancer Risk Index for gastric carcinoma

The Cancer Risk Index [22] describes important histological factors involved in promoting gastric cancerogenesis in humans, such as an increased inflammation in the corpus (corpus dominant active and chronic gastritis) as well as metaplastic changes of the mucosa. Applying this index to the gerbil model, we observed that at 16 weeks of WT-infection 30% of the animals revealed an increased carcinoma risk index. This tendency further increased up to 75% at 64 weeks of infection. In contrast, most of the mutant-infected animals did not fulfil these criteria (Table 1). Therefore, mainly *H. pylori* type I-strains with a functional T4SS are able to induce precancerous conditions, such as a corpus dominant atrophic gastritis and metaplastic changes in a gerbil stomach.

### Proinflammatory cytokine mRNA expression in antral and corpus tissue of WT-infected gerbils is drastically increased after four weeks of infection

**Figure 5:**
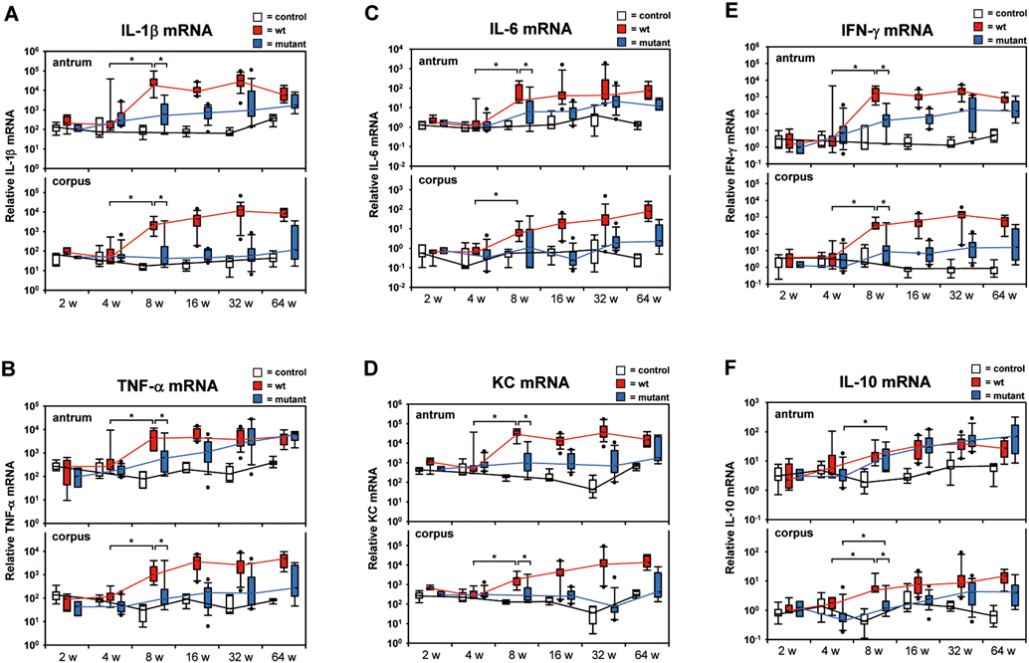
Early inflammatory events are reflected on mRNA level. (A) IL-1β, (B) TNF-α, (C) IL-6, (D) KC, (E) IFN-γ, and (F) IL-10 mRNA were measured by real-time RT-PCR normalized to 18S ribosomal RNA of antral and corpus mucosa of gerbils orally challenged with *H. pylori* B128 WT (red box) and B128Δ*cag*Y (blue box) isogenic mutant strain for 2, 4, 8, 16, 32, and 64 weeks compared with age-matched non-infected controls (white box). All box plots show 25th to 75th percentiles (box) and 5th to 95th percentiles (whiskers). Solid dots are outliers below 5% and above 95%. The line in the box represents the median. The interpolated lines connect the medians of the respective time-points. (*p<0.05).

The histological observed infiltration of inflammatory cells into the Lamina propria due to *H. pylori* infection was expected to be triggered by proinflammatory cytokines. To assess the level of cytokine mRNAs transcribed in the gastric tissue over the time course of infection, the cytokines IL-1β, TNF-α, IL-6, KC (IL-8 homologue), and IFN-γ were quantified by real time RT-PCR. Between four and eight weeks of WT-infection, the proinflammatory cytokine mRNA levels increased significantly in the antrum and corpus mucosa and remained relatively stable in the antrum until 64 weeks, but continually increased in the corpus up to 16 weeks (Figure 5A–E). Compared to basic expression levels in the non-infected control animal group the *H. pylori* WT-associated proinflammatory cytokine expression levels were induced about 25- to 200-fold in case of TNF-α, IL-6, KC, and IL-1β and even 700-fold for IFN-γ.

The mutant-infected animals revealed a gradual increase of these cytokine mRNAs in the antrum up to 32 or 64 weeks of infection. In general, the mRNA levels were about one log stage below that of WT levels, except for TNF-α. Interestingly, all analyzed proinflammatory cytokine mRNAs in the corpus of mutant-infected gerbils were comparable to the non-infected control groups, i.e. expressed basic levels over the whole infection period (Figure 5A–E).

In summary, the significant increase of cytokine mRNAs observed in antrum and corpus of WT-infected gerbils after 4 weeks clearly indicates a time-controlled *cag*-PAI-dependent mechanism of cytokine induction. At 8 weeks of infection there was a significant difference of mRNA levels in antral tissue between WT- and mutant-infected gerbils equalizing over time. These data suggest a two phase mechanism of cytokine induction in the antrum, an early *cag*-PAI dependent and a later *cag*-PAI-independent increase of proinflammatory cytokine mRNA expression by *H. pylori*.

### IL-10, an anti-inflammatory cytokine, gradually increases over time of infection

To keep a chronic inflammation running, there has to be a balance of pro- and anti-inflammatory cytokines. The anti-inflammatory cytokine IL-10 is produced by T lymphocytes and regulatory T cells and is able to attenuate the inflammation of the gastric mucosa. Interestingly, the B128 type I-strain with a functional T4SS as well as the Δ*cag*Y-mutant strain, both were able to gradually induce IL-10 mRNA in the antral and corpus mucosa during the time course experiment (Figure 5F). In contrast to the proinflammatory cytokines, the antral IL-10 mRNA level of WT-infected gerbils was not significantly induced after 4 weeks of infection, but constantly increased up to 15-fold after several months of infection. Whereas a significant increase of IL-10 mRNA expression was observed after 4 weeks infection in the corpus of WT-infected as well as in antrum and corpus of mutant-infected gerbils (Figure 5F). In conclusion we assume that the attenuated induction of an anti-inflammatory cytokine in the antral mucosa of WT-infected animals correlates with the significant increase of proinflammatory cytokines at 8 weeks of infection. This indicates IL-10 as a pivotal regulator of the *H. pylori*-induced chronic inflammation of the gastric mucosa.

### Hypergastrinemia is following hypochlorhydria after 16 weeks of infection only in WT-infected gerbils

**Figure 6:**
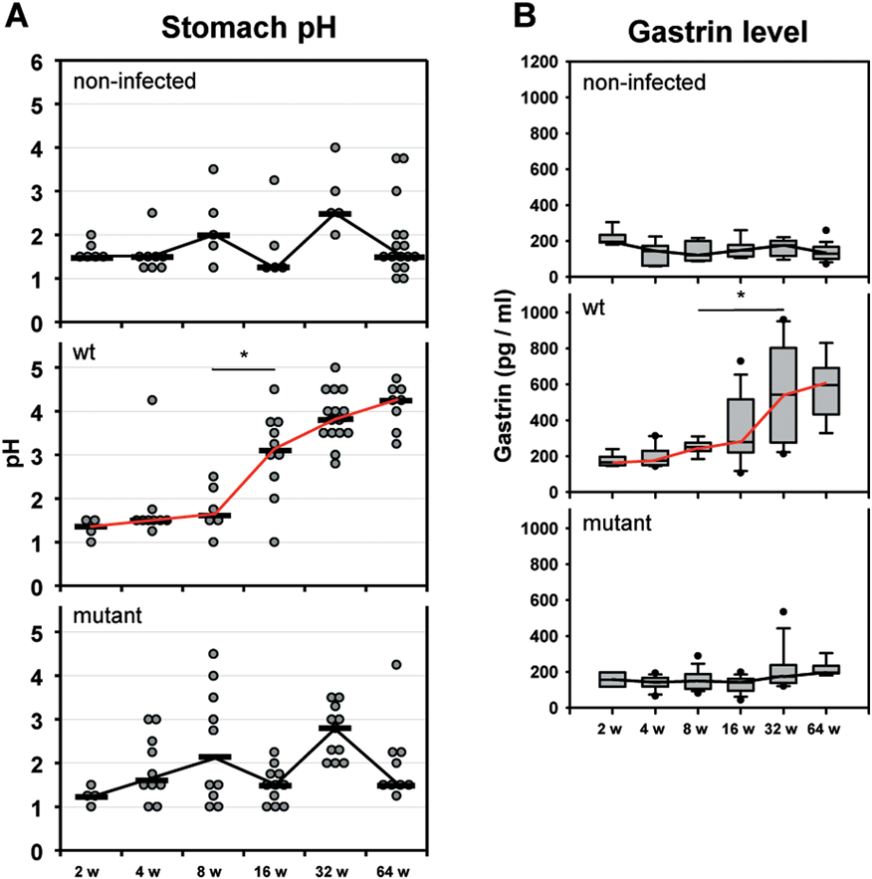
Early inflammatory events cause later physiological changes in *H. pylori* B128 WT-infected gerbils. The (A) pH and the (B) plasma gastrin level (detection limit <1 pmol/L) were determined of gerbils orally challenged with *H. pylori* B128 WT (middle panels) and B128Δ*cag*Y (bottom panels) isogenic mutant strain for 2, 4, 8, 16, 32, and 64 weeks compared with age-matched non-infected controls (upper panels). (*p<0.05).

The effect of *H. pylori*-infection on physiological parameters (pH, gastrin) in the stomach was analyzed over time. In the control animals the gastric mucosa pH was on an average of pH 1.75. However in WT-infected gerbils, a significant increase of the pH value by 1.5 units was measured at 16 weeks. The pH values continued to rise gradually to pH 4.25 at 64 weeks of infection (Figure 6A). In contrast, the mutant-infected Mongolian gerbil stabilized their gastric pH during the long term infection at the level of the control group.

Since gastrin expression is regulated by the gastric pH, we expected an increase of the gastrin level as a direct consequence of the pH changes. In fact, the WT-infected animals revealed a significant increase of plasma gastrin level at 32 weeks following a rise of the pH at 16 weeks of infection (compare Figure 6A–B). In contrast, the non-infected control group and the B128Δ*cagY*-infected animals showed very similar gastrin levels over time with a median of about 160 pg/ml. The data clearly show that the *H. pylori* WT-infection induces a relatively early increase of inflammatory markers (between 4 and 8 weeks) but a relatively late (at 16 and 32 weeks) increase in physiological parameters such as the gastric pH and the plasma gastrin level. Therefore we postulate that only an infection with a *H. pylori* type I-strain, carrying a functional T4SS able to interact with the host cells as well as translocate CagA and possibly other factors into the gastric cells, is a prerequisite to induce hypochlorhydria and hypergastrinema in the Mongolian gerbil model.

### Gastric hormone mRNA concentrations are variably changed in type I *H. pylori* infections

Several gastric hormones are regulating the homoeostasis of the stomach. Gastrin produced in G-cells located in the antral stomach as well as histamine produced by the enzyme histidine decarboxylase in enterochromaffin-like (ECL) cells are inducing the acid secretion of parietal cells in the corpus mucosa, whereas somatostatin released from D-cells in the antral and corpus mucosa is an antagonist to gastrin and histamine down-regulating the acid secretion.

**Figure 7:**
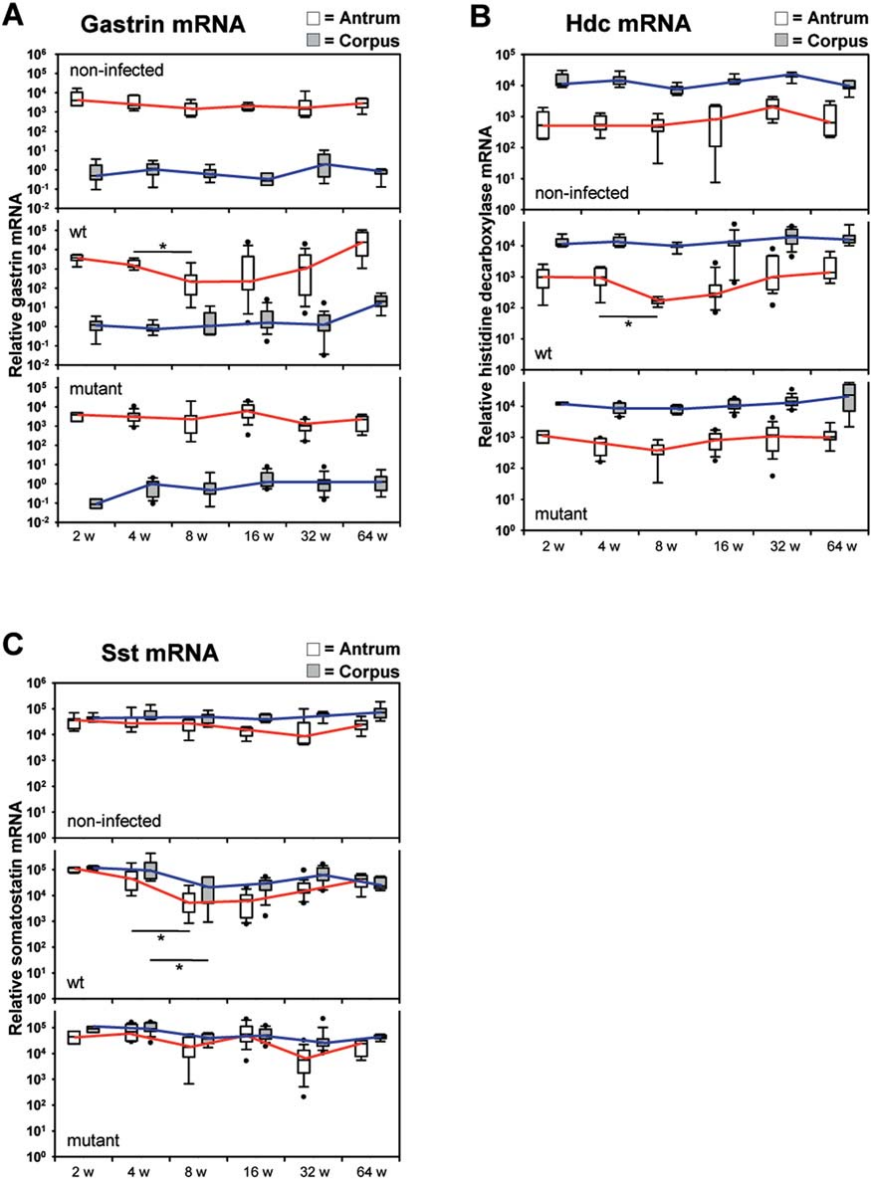
Decrease in gastrin-, histidine decarboxylase-, and somatostatin mRNA due to the *H. pylori* B128 WT-infection. (A) Gastrin-, (B) histidine decarboxylase-, and (C) somatostatin mRNA were measured by real-time RT-PCR normalized to 18S ribosomal RNA of antral (white box) and corpus (gray box) mucosa of gerbils orally challenged with *H. pylori* B128 WT (middle panels) and B128Δ*cag*Y (bottom panels) isogenic mutant strain for 2, 4, 8, 16, 32, and 64 weeks compared with age-matched non-infected controls (upper panels). All box plots show 25th to 75th percentiles (box) and 5th to 95th percentiles (whiskers). The line in the box represents the median. Solid dots are outliers below 5% and above 95%. The interpolated lines connect the medians of the respective time-points. (*p<0.05).

The gastrin and histidine decarboxylase mRNA concentration analyzed by real time RT-PCR decreased after 4 weeks significantly in antral tissue of WT-infected gerbils, but the concentrations in the corpus were constant over time (Figure 7A–B). Interestingly, the observed reduction was gradually reversed after 16 weeks of infection. A further increase of gastrin mRNA levels by one log stage was obtained at 64 weeks of infection. However, for somatostatin a significant decrease of mRNA levels was measured not only in antral, but also in corpus mucosa after 4 weeks of infection (Figure 7C). Measuring the plasma somatostatin concentration by RIA, there was a reduced plasma somatostatin concentration between 8 and 64 weeks of infection in WT-infected gerbils (data not shown).

Taken together our data reveal in WT-infected gerbils an early *cag*-PAI dependent down-regulation of hormones or enzymes involved in gastric homoeostasis on mRNA level, concomitantly with the early inflammation. However, those mRNA data are not directly correlating with the pH values and plasma gastrin levels observed after 16 weeks of infection. Thus, regulation of mRNA and protein levels is apparently separated by a time gap of several months and therefore possibly induced by two different mechanisms.

## Discussion

The pathogenesis of *H. pylori*-associated gastritis involves immunological, histological, and physiological changes in the gastric mucosa. To get a better understanding about the role of *H. pylori* and its special virulence factors on the outcome of gastric disease, we used the Mongolian gerbil model in a long term infection experiment (2–64 weeks). This animal model was chosen instead of the mouse model, since a *H. pylori* type I-strain is instable in the mouse and rapidly switches off the *cag*-T4SS.

Our data demonstrate convincingly that an intact *cag*-PAI and thus, a functional T4SS is responsible for nearly all of the observed pathological changes in the animals. The T4SS is essential for the induction of an early and severe corpus inflammation, associated with an increased expression of cytokines and histopathological changes. These cytokines seem to operate in a regulatory way on the physiology of the stomach, resulting in severe histological de-differentiation of the gastric mucosa towards the intestinal type. Therefore, this study elucidates mucosal atrophy and metaplasia as parts of the early pathomechanism due to *H. pylori* infection. We assume that an early reprogramming of gastric target cells via the *cag*-T4SS, probably by bacteria-host cell interaction and/or translocation of virulence factors, is responsible for a number of gastric changes. These are starting with an induction of severe chronic and atrophic gastritis, induction of pro-inflammatory cytokine gene transcription, finally leading to hypochlorhydria and hypergastrinemia, and late severe or malignant gastric diseases. The Mongolian gerbil model turns out to be an excellent model, to study these effects in detail. It very well mimics the human situation, with the advantage to interfere at defined stages of the process in order to eventually prevent these diseases. In a recent publication Ohnishi *et al.* revealed that a transgenic expression of *H. pylori* CagA induces gastrointestinal neoplasms in mouse [23] which supports our *in vivo*-data.

The difference in colonization density of *H. pylori* WT-strain in the stomach of Mongolian gerbils during the time course experiment revealed two *cag*-PAI dependent procedures: first, a reduced *H. pylori* density in antral mucosa and second, an increasing density in corpus mucosa, which both equalize after 32 weeks of infection. The first scenario might be explained by the development of gastric ulcers in the antrum, which correlates reciprocally with the density of the type I-strain in the gerbil stomach (Figure 1A), since *H. pylori* is unable to colonize ulcerous tissue. The second mechanism, explaining the procedure in the corpus, indicates that *H. pylori* type I-strain is continuously able to increase its load in parallel to the up-rise of the pH in the corpus mucosa of WT-infected gerbils. This reflects the destruction of parietal cells (atrophy), which is a *cag*-PAI dependent process.

Following the time course of our study, the first outstanding time point was between 4 and 8 weeks of infection. During these four weeks, the WT-infected gerbils presented already macroscopically an enlargement of the stomach size. At this early time point we assume that the translocated CagA might be active in inhibiting apoptosis, a process described recently in the Mongolian gerbil model *in vivo* [24].

Applying the grading system of Garhart *et al.* [21], a significant increase of inflammation and histological changes were already seen after four weeks of *H. pylori* type I-infection. Interestingly, at 8 weeks 100% of the infected animals developed numerous lymphoid aggregates and erosions, as well as an atrophy of the parietal cells and mucous gland metaplasia. These early observations are essential steps of the cancer pathway defined by Correa [25] indicating that a functional T4SS is a pivotal risk factor for developing gastric cancer. Some scientists argue that gastric atrophy appears to be a better indicator of gastric cancer risk than intestinal metaplasia [26]. Patients with gastric cancer of the intestinal type demonstrated a prevalence of atrophic gastritis (loss of oxyntic glands containing parietal cells) of 100% [27] that is not associated with intestinal metaplasia, but strongly associated with a mucous metaplasia (pseudopyloric metaplasia), and therefore a further important precancerous stage [28]. These observations are in accordance with our data presented here. A possible explanation is that the loss of parietal cells leads to a reduction of the secreted signal peptides that modulate the growth and differentiation of gastric progenitor cells, finally resulting in an increased proliferation of undifferentiated cells [26].

In contrast to the WT-infected animals, the mutant-infected gerbils, showed only a gradually increasing inflammation in the antral mucosa and minor histological changes (mainly erosions) over time. These changes are independent of the *H. pylori cag*-PAI. Thus, our data strongly support that a corpus predominant gastritis with a marked atrophy, as seen only in WT-infected gerbils, is associated with an increased risk for developing gastric adenocarcinoma [4].

We and others have shown that a hypochlorhydria is associated with a hypergastrinemia in *H. pylori* WT-infected animals, since gastrin is regulating the acid homeostasis in the stomach via histamine in response to an alkaline environment [17], [26], [29]. A complete atrophy of parietal cells in the corpus mucosa, in parallel to a stable G-cell differentiation in the antrum of the WT-infected gerbils supports the release of gastrin, resulting in a hypergastrinemia [30]. Therefore, it can be assumed that an important role of *H. pylori* in gastric carcinogenesis is the induction of atrophic gastritis, resulting in hypochlorhydria.

Gastrin over-expressed in an insulin-gastrin transgenic mouse model (INSGAS) induces corpus atrophy in those mice, spontaneously progressing to gastric adenocarcinoma with advanced age [31], [32]. Furthermore, a parallel infection of those mice with *Helicobacter* spp. synergises with the effect of hypergastrinemia to accelerate the malignant transformation [33]. We assume that a strong interaction of an early induced atrophy of the parietal cells together with a significantly increased stimulation of the G-cells in the antrum to release gastrin after 32 weeks of WT-infection, is responsible for severe precancerous transformations of the gastric cells. Thus, after 64 weeks of infection, gerbils challenged with a *H. pylori* type I-strain developed precancerous gastric changes, resulting in a statistically significant increase of gastritis cystica profunda (75%) and focal dysplasia (25%). Furthermore, WT-infected animals revealed an increased carcinoma risk index [22] as compared to the mutant-infected ones, but none of the infected gerbils developed gastric adenocarcinoma. Our data are in agreement with several studies of western research groups [7], [34], but contradicting published data from Japanese groups and others, that demonstrated a *H. pylori*-induced gastric cancer in Mongolian gerbils [16], [35], [36]. Thus, our data support a multifactorial process of gastric cancer induction, as it is suggested to occur in humans.

*H. pylori* induces a Th1 immune response characterized by an infiltration of T-cells releasing proinflammatory cytokines [37]. The *H. pylori* colonization of the gastric mucosa results in expression of proinflammatory cytokines such as TNF-α, IL-1β, IL-8, and IFN-γ, which attract leukocytes infiltrating the Lamina propria. This early rise of an inflammatory response is specific for a *H. pylori* type I-strain, whereas a late *cag*-PAI independent mechanism in the antral mucosa was described in mutant-infected gerbils, too. An IFN-γ null mouse challenged with *H. pylori* shows no inflammatory response, even after more than one year of infection [38]. Thus, it has been suggested that those released proinflammatory cytokines, but especially IFN-γ, possess a pivotal role in triggering cellular changes that contribute to gastric mucosal damage [38], [39]. Our data support this observation for early time points of 4 to 8 weeks of infection with a *H. pylori* type I-strain. But how does an early rise of cytokines relate to the later physiological and histological changes seen in WT-infected gerbils? We could show that the chronic gastritis is a precursor of the active gastritis in WT-infected gerbils (Figure 2B), since a mild inflammatory response was already observed by infiltrating of IFN-γ expressing T-cells after two weeks of infection. IFN-γ itself stimulates neutrophils to release proinflammatory cytokines such as TNF-α, IL-1β, and KC. Those cytokines together with IFN-γ stimulate the G-cells in the antrum to release the hormone gastrin, but they also inhibit D-cells to express somatostatin, an inhibitor of gastrin-stimulated acid secretion. [7], [40] IL-1β and TNF-α are potent inhibitors of parietal cells blocking acid secretion [41], and IL-1β decreases histamine release from enterochromaffin-like (ECL-) cells [42]. Furthermore, it was shown that TNF-α induces apoptosis of parietal cells [43] and gastrin stimulates the hyperproliferation of the gastric epithelial cells, [44] thus an enhanced inflammation, as induced by a functional *cag*-PAI positive *H. pylori*-strain, is responsible for the progression of gastric preneoplastic lesion, such as atrophy and dysplasia, to adenocercinoma [45]. In humans, carrying a *H. pylori* CagA-seropositive infection, an elevated IFN-γ concentration was also detected that accounts for an increased plasma gastrin level [46].

In contrast, the anti-inflammatory Th2 cytokine IL-4 stimulates the secretion of somatostatin [47], thereby decreasing the development of gastric atrophy [48]. *H. pylori* type II-strain infected gerbils revealed a significant increase of the anti-inflammatory cytokine IL-10 mRNA in antral and corpus tissue after four weeks of infection whereas, gerbils challenged with the type I-strain only gradually increased the IL-10 mRNA level in antral mucosa without any significance. Due to the reduced IL-10 expression in WT-infected gerbils, the Th1/Th2 equilibrium, which sustains a chronic inflammation, is deranged resulting in a hypergastrinemia and atrophy of the parietal cells in the oxyntic mucosa. Therefore, our results indicate a *H. pylori* type I-strain dependent early modulation of the immune response towards a Th1 response that is dominated by an IFN-γ regulating cascade. This includes the stimulation of further proinflammatory Th1 cytokines that in the following regulate the gastric physiology resulting in severe histological changes such as precancerous lesions.

In conclusion, the Mongolian gerbil model is the only suitable rodent model available, mimicking the human situation, for analyzing the effect of major *H. pylori* virulence factors on the pathogenesis of the *H. pylori*-infected gastric mucosa. As our study reveals, we could standardize the gerbil model for investigating the complex interaction of immunological, physiological, and histological parameters in antral and corpus mucosa separately during a long-term *H. pylori*-infection experiment. Further clinical aspects of the pathogenesis of *H. pylori* towards gastric adenocarcinoma can now be addressed. In future, these results might be of therapeutic relevance.

## Supporting Information

Table S1 (0.08 MB DOC)
