# Supplementary material for: Helicobacter pylori cag-Pathogenicity Island-Dependent Early Immunological Response Triggers Later Precancerous Gastric Changes in Mongolian Gerbils
Source: PLoS One. 2009 Mar 9;4(3):e4754. doi: 10.1371/journal.pone.0004754 (PMC2650263; doi:10.1371/journal.pone.0004754)
Supplement: Table S1 — (0.08 MB DOC) [file pone.0004754.s001.doc]

| **Table S1** |  |  |  |  |
| --- | --- | --- | --- | --- |
| **Oligonucleotide primers and probes for sequence detection and real-time RT-PCR** | | | | |
|  |  | **IL-6** |  |  |
| **detection primers** | |  |  |  |
|  |  |  |  |  |
| forward primer | | 5`-CCTCTTGTTTGAAGATACGAATTA-3` | | |
| reverse primer | | 5`-GCTGATGGTGGTGATGGCCA-3` | | |
|  |  |  |  |  |
| **real-time primers and probes** | | |  |  |
|  |  |  |  |  |
| forward primer | | 5’-TGAAACTTCCAGAGATACAGAGAGATGA-3’ | | |
| reverse primer | | 5’-CAGAAGACCAGAGGTGATTTTCAAT-3’ | | |
| probe |  | 5’-6FAM-TTCCATACTGGATACAATCGGGACGTTTGC-XT-PH-3’ | | |
|  |  |  |  |  |
|  |  | **IL-10** |  |  |
| **detection primers** | |  |  |  |
|  |  |  |  |  |
| forward primer | | 5`-GCCCAGCAGAGGCGAGTAC-3` | | |
| reverse primer | | 5`-CCAAGGAGGTGCTTCTGTTAG-3` | | |
|  |  |  |  |  |
| **real-time primers and probes** | | |  |  |
|  |  |  |  |  |
| forward primer | | 5’-AAGTTGAAGACCCTCAGAATGCA-3’ | | |
| reverse primer | | 5’-CACAGCCTTGCTCTTATTTTCACA-3’ | | |
| probe |  | 5’-6FAM-CTGCGGCGCTGTCATCGATTTCTC-XT-PH-3’ | | |
|  |  |  |  |  |
|  |  | **gastrin** |  |  |
| **detection primers** | |  |  |  |
|  |  |  |  |  |
| forward primer | | 5`-GGAAGCCCCGCTCCCAGCTACAGGATG-3` | | |
| reverse primer | | 5`-TCCGTGGCCTCTGCTTCTTGGACAGGTC-3` | | |
|  |  |  |  |  |
| **real-time primers and probes** | | |  |  |
|  |  |  |  |  |
| forward primer | | 5’-GCCCTGGAACCGCAACA-3’ | | |
| reverse primer | | 5’-TTCTTGGACAGGTCTGCTTTGAA-3’ | | |
| probe |  | 5’-6FAM-AGCTGGGCCTAACCTCTCACCATCGAA-XT-PH-3’ | | |
|  |  |  |  |  |
|  |  | **histidine decarboxylase** | | |
| **detection primers** | |  |  |  |
|  |  |  |  |  |
| forward primer | | 5`-GGTCAAGGACAAGTACAAGC-3` | | |
| reverse primer | | 5`-GACAGCTTGTGCTTGGTGG-3` | | |
|  |  |  |  |  |
| **real-time primers and probes** | | |  |  |
|  |  |  |  |  |
| forward primer | | 5’-GACTTCATGCATTGGCAGATCC-3’ | | |
| reverse primer | | 5’-CACCCCGAAGGACCGAAT-3’ | | |
| probe |  | 5’-6FAM-AGCCGGCGCTTTCGCTCCATTAA-XT-PH-3’ | | |
|  |  |  |  |  |
|  |  | **TNF-α** |  |  |
| **detection primers** | |  |  |  |
|  |  |  |  |  |
| forward primer | | 5`-GGCCCAGACCCTCACACTC-3` | | |
| reverse primer | | 5`-GGGAGTACACGAGGTACAGC-3` | | |
|  |  |  |  |  |
| **real-time primers and probes** | | |  |  |
|  |  |  |  |  |
| forward primer | | 5’-CACACTCAGGTCCTCTTCTCAGAAC-3’ | | |
| reverse primer | | 5’-CAGCTGCTCCTCCACTTGGT-3’ | | |
| probe |  | 5’-6FAM-AGCCTGTGGCCCATGTCGTAGCC-XT-PH-3’ | | |
|  |  |  |  |  |
